# Supplementation of Oocytes by Microinjection with Extra Copies of mtDNA Alters Metabolite Profiles and Interactions with Expressed Genes in a Tissue-Specific Manner

**DOI:** 10.3390/biom14111477

**Published:** 2024-11-20

**Authors:** Eryk Andreas, Alexander Penn, Takashi Okada, Justin C. St. John

**Affiliations:** Experimental Mitochondrial Genetics Group, School of Biomedicine, Faculty of Health and Medical Sciences, The University of Adelaide, Adelaide Health and Medical Sciences Building, Adelaide, SA 5000, Australia; eryk.andreas@adelaide.edu.au (E.A.); alexander.penn@adelaide.edu.au (A.P.); takashi.okada@adelaide.edu.au (T.O.)

**Keywords:** mtDNA, mtDNA supplementation, metabolite profile, gene expression, metabolic pathways, brain, liver, heart

## Abstract

Mitochondrial DNA (mtDNA) supplementation can rescue poor oocyte quality and overcome embryonic arrest. Here, we investigated a series of sexually mature pigs generated through autologous and heterologous mtDNA supplementation. Brain, liver and heart tissues underwent metabolite profiling using gas chromatography–mass spectrometry and gene expression analysis through RNA-seq. They were then assessed for mRNA–metabolite interactions. The comparison between overall mtDNA supplemented and control pigs revealed that mtDNA supplementation reduced the lipids stearic acid and elaidic acid in heart tissue. However, heterologous mtDNA supplemented-derived pigs exhibited lower levels of abundance of metabolites when compared with autologous-derived pigs. In the brain, these included mannose, mannose 6-phosphate and fructose 6-phosphate. In the liver, maltose and cellobiose, and in the heart, glycine and glutamate were affected. mRNA–metabolite pathway analysis revealed a correlation between malate and *CS*, *ACLY*, *IDH2* and *PKLR* in the liver and glutamate and *PSAT1*, *PHGDH*, *CDO1* and *ANPEP* in the heart. Our outcomes demonstrate that mtDNA supplementation, especially heterologous supplementation, alters the metabolite and transcriptome profiles of brain, liver, and heart tissues. This is likely due to the extensive resetting of the balance between the nuclear and mitochondrial genomes in the preimplantation embryo, which induces a series of downstream effects.

## 1. Introduction

There is increasing demand to use assisted reproductive technologies to help couples to have their own genetic children. However, in a number of cases, their oocytes are depleted of mitochondrial DNA (mtDNA) [1]. They can be rescued through supplementation with extra copies of mtDNA at the time of fertilisation [2] and can give rise to live offspring [3]. However, the impact on tissue-specific metabolomics and gene expression needs to be investigated in order to determine if the process impacts the offspring’s health and well-being.

The maternally inherited [4] mitochondrial genome is double-stranded, circular and approximately 16.6 kb in size [5]. It comprises 13 genes that encode subunits of the electron transfer chain, 22 tRNAs, and two rRNAs and has one major non-coding region, namely the displacement loop (D-loop), which is the site of interaction for the nuclear-encoded mtDNA transcription and replication factors. mtDNA copy number ranges from several in spermatozoa [6] to several hundred thousand in mature oocytes [1].

The maintenance of mtDNA copy number is essential for normal cell function, especially during development [7,8]. Indeed, there appears to be a threshold of mtDNA copy number that needs to be surpassed for successful fertilisation and subsequent preimplantation embryonic developmental outcome, as demonstrated in humans and pigs [1,9]. To this extent, fertilisation of mtDNA-deficient metaphase II (MII) oocytes, which possess as few as ~50,000 copies of mtDNA, resulted in either failed fertilisation or very poor rates of development to the blastocyst stage, the final stage of preimplantation development. However, supplementation of mtDNA-deficient oocytes with extra copies of mtDNA as they were fertilised by intracytoplasmic sperm injection (ICSI) significantly improved fertilisation outcome and development to the blastocyst [2]. The blastocysts exhibited gene expression profiles, networks, and pathways that were more similar to blastocysts derived from oocytes with the requisite numbers of mtDNA copies.

Supplementation of MII oocytes containing requisite levels of mtDNA with extra copies isolated from sister (autologous) oocytes resulted in the differential methylation of over 2000 genomic regions and the differential expression of 52 genes by the blastocyst stage [10]. In both mtDNA-deficient and non-deficient oocytes, approximately 780 copies of mtDNA were introduced, which represents 0.39% of a normal oocyte’s mtDNA copy number. Consequently, mtDNA supplementation does not increase the number of mitochondria but increases mtDNA content relative to nuclear DNA. This requires the two genomes to reset their ‘genomic balance’ to mediate cooperation, as shown in tumour [11,12] and stem cell models [13,14]. Indeed, the balance between the two genomes is likely established during oogenesis as the primordial germ cells differentiate into mature oocytes and undergo waves of de/methylation [15] coupled with significant increases in mtDNA copy. This provides mature oocytes with fertilisation capacity and the requisite number of copies of mtDNA [9] to sustain early embryonic development when there is little or no replication of mtDNA until post-gastrulation [7,8].

In addition, the effects of the segregation of wild-type and mutant copies of mtDNA (heteroplasmy) are still unclear [16] although ~60% of mutant mtDNA can mediate the onset of mtDNA diseases [17]. However, a recent study in mice revealed that the presence of another non-pathogenic mtDNA variant (divergent non-pathogenic mtDNA heteroplasmy), which potentially occurs in mitochondrial-related therapies including mtDNA supplementation and mitochondrial replacement therapy, impaired mitochondrial function, induced cell metabolic stress and led to cell death in the tissues of adult mice [18].

mtDNA supplementation offers the opportunity to study three key areas. Firstly, it enables the study of how the mitochondrial genome can influence the nuclear genome in a model that is dynamic by looking at endpoint tissues [19]. Secondly, it can determine how mtDNA supplementation in the oocyte influences offspring health and well-being and whether the outcomes are applicable to assisted reproduction for couples that suffer from failed fertilisation or repeated preimplantation embryo developmental failure [19]. Finally, it acts as a model to understand the effects of mtDNA carryover in the context of mitochondrial donation as a proposed treatment to prevent the transmission of mtDNA [20] or as a more aggressive, invasive approach to treating fertilisation failure and embryonic arrest [21]. In this derivative of nuclear transfer, a small amount of mtDNA is carried over with the karyoplast into the donor oocyte [21]. Consequently, mtDNA supplementation allows the effect of mtDNA carryover to be investigated in isolation without the confounding factors associated with the karyoplast adjusting to its new cytoplasmic environment.

We have previously generated porcine offspring using mtDNA derived from sister (autologous) or third-party (heterologous) oocytes [19]. The piglets reached sexual maturity but exhibited some differences in growth and biochemical and haematological parameters [19] and differences in gene expression in brain, liver and heart tissue [22]. Consequently, we hypothesised that the extra mtDNA would alter the metabolomic profiles of these tissues. In this respect, we have identified metabolic changes resulting from mtDNA supplementation in adult brain, liver and heart tissues relative to control animals. We then determined if there were any differences in metabolite and gene expression profiles between autologous and heterologous derived tissues and if specific pathways were enriched. Finally, we determined if there were interactions between the differences in metabolites and gene expression in these tissues. Overall, mtDNA supplementation changed a number of metabolites compared to controls. However, there were a greater number of differentially relatively abundant metabolites in brain, liver and heart tissue between the heterologous and autologous cohorts. Those metabolites were mostly involved in carbohydrate metabolism in the brain and liver and protein metabolism in the heart. By combining gene expression and metabolite analyses, the citrate cycle (tricarboxylic acid cycle-TCA cycle) and glycine, serine and threonine metabolism pathways were significantly affected by heterologous supplementation in the liver and heart, respectively.

## 2. Materials and Methods

Unless mentioned otherwise, all chemicals and consumables were supplied by Merck Life Science Pty Ltd. (Bayswater, VIC, Australia).

### 2.1. Cumulus-Oocyte-Complexes (COCs) Isolation and In Vitro Maturation

Pairs of ovaries collected from gilts at a local abattoir were transported to the laboratory in a vacuum flask containing warm 0.9% NaCl solution (Baxter, Old Toongabbie, NSW, Australia). The COCs were aspirated from healthy Graafian follicles exhibiting a diameter of 3 to 6 mm using an 18 G needle. COCs from each ovary pair were kept together. The COCs were then washed three times using handling media consisting of 25 mM Hepes-TCM199 media (Thermo Fisher Scientific Australia, Scoresby, VIC, Australia) supplemented with 10% sow follicular fluid (SFF). Healthy COCs were selected and cultured in individual wells for 42 to 44 h in 500 μL pre-equilibrated in vitro maturation (IVM) media, which comprised TCM199 media (Thermo Fisher Scientific Australia) supplemented with 0.80 mM Na-pyruvate, 0.61 mM L-glutamine, 0.88 M cysteamine, 5 μg/mL insulin, 10 IU/mL PMSG (Folligon^®^, Pacific Vet Pty. Ltd., Braeside, VIC, Australia), 10 IU/mL hCG (Chorulon^®^, Pacific Vet Pty. Ltd.), 0.10 μg/mL EGF and 10% SFF, and maintained at 38.5 °C in a humidified incubator with 5% CO_2_ in air.

### 2.2. MII Oocyte Collection

MII oocytes were collected by mixing 0.1% (0.5 mg/mL) hyaluronidase into individual wells containing expanded COCs from each ovary pair. The remaining cumulus cells were stripped using a narrow pipette. MII oocytes, which were indicated by the presence of a first polar body, were kept in a microdroplet until required, whilst non-MII oocytes were collected for mitochondrial isolation.

### 2.3. Mitochondrial Isolation from Oocytes

Mitochondrial fractions were isolated from non-MII oocytes, as previously described [2,10,22]. Briefly, 10 to 15 oocytes were resuspended in 5 mL of mitochondrial isolation buffer (20 mM Hepes pH 7.6, 220 mM mannitol, 70 mM sucrose, 1 mM EDTA) containing 2 mg/mL BSA. The oocytes were then homogenised by 10 strokes of a drill-fitted Potter-Elvehjem tissue grinder set (VWR International, Radnor, PA, USA) on ice. Cell debris was removed by centrifuging the oocyte homogenate at 800× *g* for 10 min at 4 °C. Mitochondrial supernatants were pelleted by centrifugation at 10,000× *g* for 20 min at 4 °C. The mitochondrial pellet was resuspended in 700 μL of mitochondrial isolation buffer and reconcentrated by centrifuging at 10,000× *g* for 20 min. The mitochondrial concentrate was then resuspended in 5 μL of mitochondrial isolation buffer in readiness for mitochondrial supplementation.

### 2.4. Animal Ethics Declaration

All procedures involving live animals were assessed by the University of Adelaide Animal Ethics Committee (approval number 32293). All animals were kept in a temperature-control room at 22 °C, with ad libitum access to water and feed.

### 2.5. Generation of mtDNA-Supplemented Pigs

mtDNA-supplemented pigs were generated by injecting purified populations of mitochondria containing ~780 copies of mtDNA into the oocyte at the time of ICSI. This procedure involved preloading the microinjection pipette with 3 ρl of mitochondrial isolate and a single spermatozoon and injecting both simultaneously into a denuded MII oocyte. Prior to injection, the MII oocyte was held with the polar body orientated at 6 or 12 o’clock, while the sperm injection pipette was inserted at 3 o’clock opposite the holding pipette, which was at 9 o’clock. This positioning avoided spindle damage due to injection or suction. Putative zygotes were then transferred into oestrus-synchronised post-pubertal Large White x Landrace gilts at 26 weeks of age as recipients for embryo transfer (ET), as described in [19]. Meanwhile, for the control group, the post-pubertal gilts were allowed to mate naturally with a colony boar. Pregnancy was determined by ultrasound between days 28 and day 45 post-ET through the presence of visible amniotic vesicles. Surrogates were housed in pens until 4 days prior to expected farrowing and then transferred into a farrowing crate. After weaning (at 28 d) until 10 weeks of age, piglets were fed with a commercial weaner diet, whilst for weeks 10 to 20, piglets were fed with a commercial grower diet. Starting at 20 weeks of age, they were fed with a finisher diet.

### 2.6. Tissue Collection

At the time of autopsy, 50–100 mg tissues from identical positions in the brain, liver and heart were collected. The samples were transferred into 2 mL cryogenic tubes (Corning^®^). The samples were snapped frozen in liquid N_2_ and then stored at −80 °C until used for gene expression and metabolite analysis. Sections from the same piece of tissue for each animal were used for metabolite and gene expression analyses.

### 2.7. Sample Preparation for Metabolomic Analysis

The sample weights were recorded before and after lyophilisation. The tissue samples were transferred into bead mill tubes and homogenised by adding 1 μL MiliQ water for each 0.1 mg of tissue and were centrifuged for 4 cycles of 60 s at 8000 rpm with 90 s pauses between cycles. Methanol (410 μL) was added to 25 μL of the sample to precipitate proteins. Ten μL of 0.095 mM L-Valine-^13^C_5_,^13^N was added to the samples. The samples were then vortexed for 30 s and centrifuged at 4 °C for 10 min at 14,000 rpm. A similar volume of supernatant from each sample was pooled into 6 pooled biological quality control (PBQC) samples. A 180 μL aliquot of supernatant from each sample and PBQC was transferred into 250 μL glass inserts in a new 1.5 mL Safe-Lock microcentrifuge tube (Eppendorf^®^) and evaporated to complete dryness at room temperature using a Centrivap (Labconco, Kansas City, MO, USA) for 3 h. Once dry, the inserts were transferred into 2 mL vials and capped for analysis.

### 2.8. Metabolomic Reading Using Gas Chromatography-Mass Spectrometry (GC-MS)

The analysis was performed on a Shimadzu GCMS-TQ8050 NX (Shimadzu Scientific, Rydalmere, NSW, Australia) equipped with an AOC-6000 Plus Multifunctional Autosampler System (Shimadzu Scientific). Instrument control was performed with Labsolutions GCMS solution 4.53 (Shimadzu Scientific). The instrument was fitted with an Agilent J&W DB-5 GC Column (30 m, 0.25 mm, 1.00 μm) with helium carrier gas. The derivatisation of the samples was performed using PAL online derivatisation. Methoxyamine hydrochloride in pyridine (22 μL) was added to the samples and incubated at 37 °C with shaking for 2 h. BSTFA + 1% TMCS (22 μL) was added, and the samples were incubated at 37 °C with shaking for 30 min. The samples were allowed to settle at room temperature for 1 h before injection.

The derivatised sample (1 μL) was injected into the inlet at 280 °C in pulsed split mode. The oven temperature was started at 100 °C (held for 4 min) and then increased to 320 °C at 10 °C/min (held for 11.81 min). The total run time was 37.81 min. The mass spectrometer quadrupole temperature was set at 200 °C, the source was set at 200 °C, and the transfer line was held at 280 °C. The samples were acquired using the multiple reaction monitoring mode. All samples were analysed in a random order with a derivatisation blank (clean insert in vial) run at the beginning of each run, while PBQCs were run at the end.

### 2.9. Metabolomic Data Analysis

Raw metabolomic data were further processed using Shimadzu LabSolutions insights (version 4.0 SP 2.0) software. Curation steps included the removal of signals that were unreliable, as indicated by the PBQC with Coefficients of Variation (%CV) above 30% and signal-to-noise below 3. Only metabolites with all values present were used for statistical analysis. Curated data were normalised using probabilistic quotient normalisation (PQN; [23]) and scaled using autoscaling methods by dividing each variable with the standard deviation of each variable in the control cohort. Mean differences between the cohorts were determined by *t*-test. Heatmap for fold change (FC) and enriched Kyoto Encyclopedia of Genes and Genomes (KEGG) pathways analysis were performed using MetaboAnalyst [24]. The metabolites were considered to be differentially relatively abundant metabolites if the *p*-value from the t-student test was <0.05 (*p* < 0.05). KEGG pathways are considered as being affected if there are two minimum hits and *p* < 0.05.

### 2.10. Differentially Expressed Genes (DEGs) and mRNA–Metabolites Interaction Network Analysis

Data for the DEG analysis were obtained from a publicly available dataset under the BioProject ID PRJNA823749, as described in [22]. DEGs between mtDNA-supplemented pigs and controls and between heterologous and autologous mtDNA-supplemented pigs were analysed using edgeR [25]. Genes are considered differentially expressed if their FDR (false discovery rate) is <0.05. The mRNA–metabolite interaction network was analysed using MetaboAnalyst [24]. The chemical and human gene associations were extracted from the search tool for interactions of chemicals (STITCH), and only subnetworks with at least 3 nodes were considered for mapping analysis.

## 3. Results

### 3.1. The Effect of mtDNA Supplementation on Metabolite Profiles in Brain, Liver and Heart Tissue

Since we found that mtDNA supplementation of the oocyte at the time of fertilisation by intracytoplasmic sperm injection (ICSI) significantly altered global DNA methylation patterns of blastocysts [10] and the mRNA profiles of resultant offspring tissues [22], we asked whether mtDNA supplementation affects the metabolite profiles of tissues. In this respect, we tested three high- Oxidative phosphorylation (OXPHOS)-requiring tissues, namely brain, liver and heart. Metabolite profiles were assessed using non-volatile polar metabolite GC-MS registered targets, and raw data were read by using Shimadzu LabSolutions insights (version 4.0 SP 2.0) software. Curated data were normalised using PQN) [23] and scaled using autoscaling methods, dividing each variable by the standard deviation of each variable in the control group (Figure S1).

For the 27 samples analysed through GC-MS, between 128 and 140 compounds were detected from a total of 598 compounds. For statistical analysis, a total of 109 compounds in the liver, 111 in the brain, and 111 compounds in the heart were used for further analysis. The list of differentially relatively abundant compounds for each tissue is presented in Table 1, and the FC are visualised in the heatmap (Figure S2). There were three differentially relatively abundant compounds (*p* < 0.05) in offspring brain tissue, namely 2-hydroxyisobutyric acid and stearic acid, that were increased as a result of overall mtDNA supplementation (FC 1.46 and 1.21, respectively), and 5-oxoproline which was lower (FC 0.86) in the mtDNA-supplemented group.

In the liver, inositol was found to be relatively higher (FC 1.25) in mtDNA-supplemented tissues compared to control (Table 1). However, eleven differentially abundant compounds were identified in pig heart (*p* < 0.05), namely ribose 5-phosphate, hypotaurine and creatinine, which were relatively lower (FC 0.73, 0.66 and 0.73, respectively), whilst N-acetylglucosamine, stearic acid, threonine, 2-hydroxyisobutyric acid, margaric acid, elaidic acid, oleic acid and monostearin (FC 1.65, 1.81, 1.48, 1.63, 1.69, 2.32, 2.29 and 1.61, respectively) were higher in mtDNA-supplemented tissues compared to controls (Table 1).

Furthermore, MetaboAnalyst 6.0 software [24] was used to compare the mean value from each treatment group and analyse enriched metabolic pathways based on outputs from the KEGG database. KEGG pathway enrichment analysis showed that mtDNA supplementation significantly (*p* < 0.05) altered glutathione metabolism and biosynthesis of unsaturated fatty acids in the brain. It also altered ascorbate and aldarate metabolism, galactose metabolism, and inositol phosphate metabolism in the liver. In the heart, biosynthesis of unsaturated fatty acids, valine, leucine, and isoleucine biosynthesis, as well as taurine and hypotaurine metabolism, were affected by mtDNA supplementation. However, mapping analysis showed only biosynthesis of unsaturated fatty acids in heart tissue to have a substantial number of compounds (minimum hits = 2 and *p* < 0.05) in their mapped pathways (Table S1), namely stearic and elaidic acid.

### 3.2. The Source of mtDNA Affects Metabolic Pathways in Brain, Liver and Heart Tissues

We hypothesised that different sources of mtDNA would affect the metabolite profiles of offspring tissues. The supplemented group was divided into two subgroups, i.e., heterologous and autologous, based on the source of mtDNA used for mtDNA supplementation. Heterologous supplementation used mtDNA isolated from oocytes from a third-party ovarian source, whilst autologous supplementation used mtDNA isolated from oocytes from the same ovarian source. Statistical analysis showed that mannose 6-phosphate (2), fructose 6-phosphate, mannose, glucose 6-phosphate, mannose 6-phosphate (1) and galactose (FC 0.31, 0.33, 0.43, 0.31, 0.31 and 0.22, respectively) were significantly lower, whilst 2-aminoethanol, ornithine, 3-phosphoglyceric acid, uracil and thymine (FC 1.59, 1.10, 3.92, 1.25 and 1.57, respectively) were significantly higher in pig brains derived from the heterologous cohort compared to the autologous mtDNA-supplemented group (*p* < 0.05; Table 2). Furthermore, fumaric acid, malic acid, arabitol, inositol, lactitol, trehalose, cellobiose and maltose (FC 0.61, 0.71, 0.48, 0.90, 0.30, 0.37, 0.37 and 0.38, respectively) were significantly lower, whilst glyceric acid, 1-hexadecanol, valine and tyrosine (FC 1.47, 1.23 1.40 and 1.41) were significantly higher in pig livers derived from the heterologous mtDNA-supplemented group (*p* < 0.05; Table 3). Lastly, in heart tissue, we found that creatinine, glycerol, fumaric acid, 3-hydroxyisovaleric acid, pantothenic acid, adenosine and lactic acid (FC 1.29, 1.70, 1.77, 2.04, 1.55, 2,18 and 1.17, respectively) were significantly higher, whilst palmitic acid, glycine, margaric acid, ribulose, glutamate, cysteine and xylulose (FC 0.63, 0.82, 0.70, 0.48, 0.58, 0.48 and 0.51, respectively) were significantly lower in abundance in the heterologous mtDNA-supplemented group (*p* < 0.05; Table 4). FC values can also be visualised in the heatmap (Figure S3).

KEGG analysis showed that several pathways were found to be significantly affected (*p* < 0.05 and minimum hits = 2) by heterologous mtDNA supplementation. In the brain, carbohydrate metabolism was affected, which included fructose and mannose metabolism, starch and sucrose metabolism, galactose metabolism, amino sugar and nucleotide sugar metabolism, and protein metabolism, including arginine biosynthesis, pyrimidine metabolism, and glutathione metabolism (Table S2). In the liver, carbohydrate metabolism was altered (Table S3). This included starch and sucrose metabolism, TCA cycle, and glyoxylate and dicarboxylate metabolism. In addition, protein metabolism was affected through tyrosine metabolism. In the heart, protein metabolism was also affected (Table S4), which included glutathione metabolism, arginine biosynthesis, pantothenate and CoA biosynthesis, alanine, aspartate and glutamate metabolism, porphyrin metabolism, and glycine, and serine and threonine metabolism. In addition, carbohydrate metabolism was also affected, including pentose and glucuronate interconversions, as well as glyoxylate and dicarboxylate metabolism.

**Figure 1:**
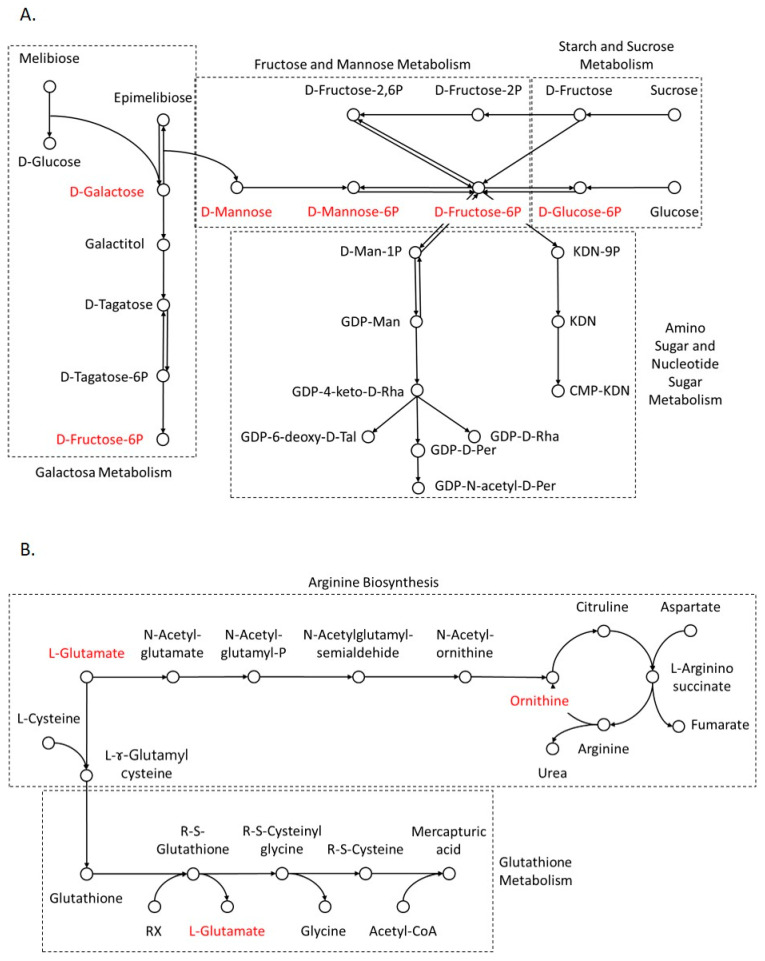
Schematic diagram of carbohydrate (**A**) and protein (**B**) metabolism in offspring brain. Red font indicates differentially relatively abundant compounds for the comparison between heterologous and autologous mtDNA-supplemented pigs.

Mapping the differentially relatively abundant compounds in brain tissue derived from heterologous compared to autologous mtDNA-supplemented pigs showed the interconnections within carbohydrate metabolism. These included fructose and mannose metabolism, starch and sucrose metabolism, galactose metabolism, and amino sugar and nucleotide sugar metabolism. As shown in Figure 1A, mannose, galactose, fructose 6-phosphate and mannose 6-phosphate were mapped in those sub-carbohydrate metabolism groups. Glucose and other simple sugars such as galactose, mannose and fructose in brain tissue play pivotal roles as the fuel source for brain cell and neuronal activity [26]. On the other hand, glutamine, glutamate and ornithine are involved in protein metabolism, including arginine biosynthesis and glutathione metabolism (Figure 1B). Protein metabolism in the brain is not limited to building protein blocks but is also involved in brain signal transmission. For instance, glutamate and glycine are known to be major inhibitory neurotransmitters [27].

**Figure 2:**
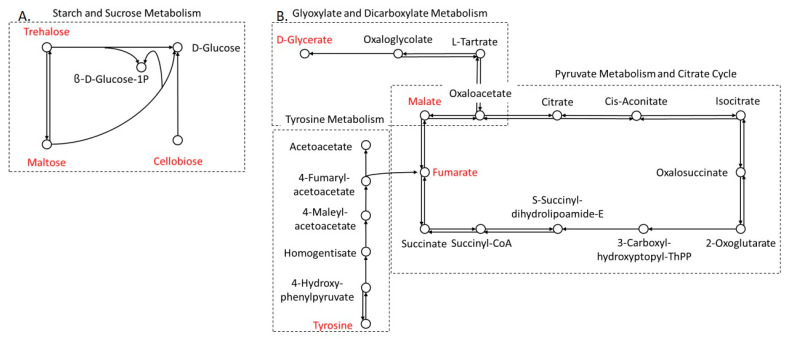
Schematic diagram of starch and sucrose (**A**) and carbohydrate–protein (**B**) metabolism in offspring liver. Red font indicates differentially relatively abundant compounds for the comparison between heterologous and autologous mtDNA-supplemented pigs.

In the liver, carbohydrate metabolism is essential for maintaining haemostatic glucose levels in the blood system by controlling the balance between glucose anabolism and catabolism [28]. Here, we found that disaccharides, including trehalose, maltose and cellobiose, were affected in carbohydrate metabolism, namely starch and sucrose metabolism (Figure 2A). In addition, there were interactions between carbohydrate and protein metabolism, glyoxylate and dicarboxylate metabolism, pyruvate metabolism, TCA cycle, and tyrosine metabolism (Figure 2B). Metabolites involving tyrosine and glycerate, which produce fumarate and malate, respectively, are used to generate adenosine triphosphate (ATP) for energy [29].

**Figure 3:**
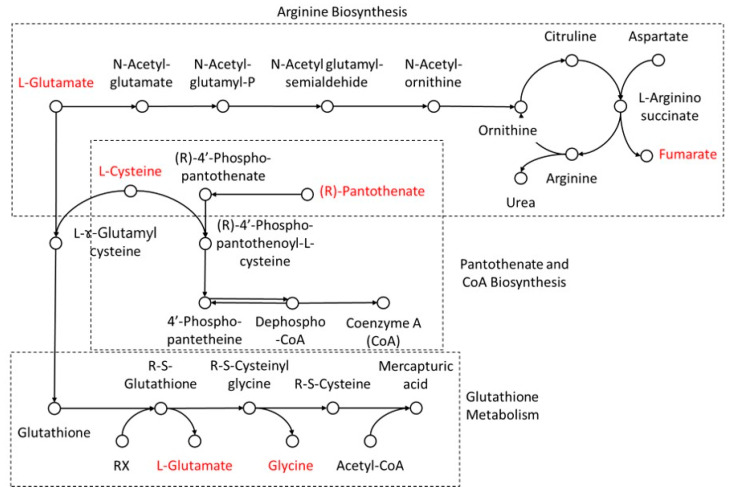
Schematic diagram of protein metabolism in offspring heart. Red font indicates differentially relatively abundant compounds for the comparison between heterologous and autologous mtDNA-supplemented pigs.

Lastly, fatty acid or lipid metabolism through β-oxidation is known to be the main source of energy for cardiomyocytes. However, excessive lipid metabolism exhibited an accumulation of reactive oxygen species (ROS), which decreases cell viability and disturbs mitochondrial function [30]. Therefore, antioxidant metabolism plays an important role in maintaining normal heart function. For example, glutathione, an endogenous antioxidant, reduces ROS accumulation [31]. Here, we found that protein metabolism, including arginine biosynthesis, pantothenate and CoA biosynthesis, and glutathione metabolism, were significantly affected by heterologous mtDNA supplementation through glutamate, glycine, cysteine, fumarate and pantothenate. As highlighted in Figure 3, cysteine and glutamate are essential for glutathione synthesis, whilst fumarate is also known as a cardioprotective substrate through activation of the NRF2 antioxidant pathway [32].

### 3.3. mRNA–Metabolite Network Analysis

In order to analyse mRNA–metabolite interactions, we combined the data from the differentially relative compounds with DEGs for each of the offspring’s tissues based on the STITCH database [33]. DEGs in the offsprings’ brain, liver and heart were reanalysed using edgeR from previously published datasets from the same tissue sources used for metabolite analyses [22]. The comparison between mtDNA-supplemented and control tissues showed that a total of 161, 15 and 9 DEGs were found in the liver, heart and brain, respectively (Tables S5–S7). This comprises 120, 4 and 1 upregulated genes in liver, heart and brain tissue, respectively. Due to the relatively few DEGs and differentially relatively abundant compounds in the comparison between the overall mtDNA supplemented and control groups, network analysis did not reveal any mRNA–metabolite networks in brain, liver and heart tissue. The list of DEGs for brain, liver and heart are presented in Tables S5–S7.

In addition, the comparison between heterologous and autologous mtDNA supplementation revealed 392, 313 and 52 DEGs in liver, heart and brain tissue, respectively, of which 228 (liver), 147 (heart) and 27 (brain) were upregulated. The list of DEGs for brain, liver and heart are presented in Tables S8–S10. Furthermore, enrichment analysis showed that the TCA cycle and pyruvate metabolism pathways were significantly (*p* < 0.05 and minimum hits = 3) affected by differential mRNA–metabolite interactions (Table S11).

**Figure 4:**
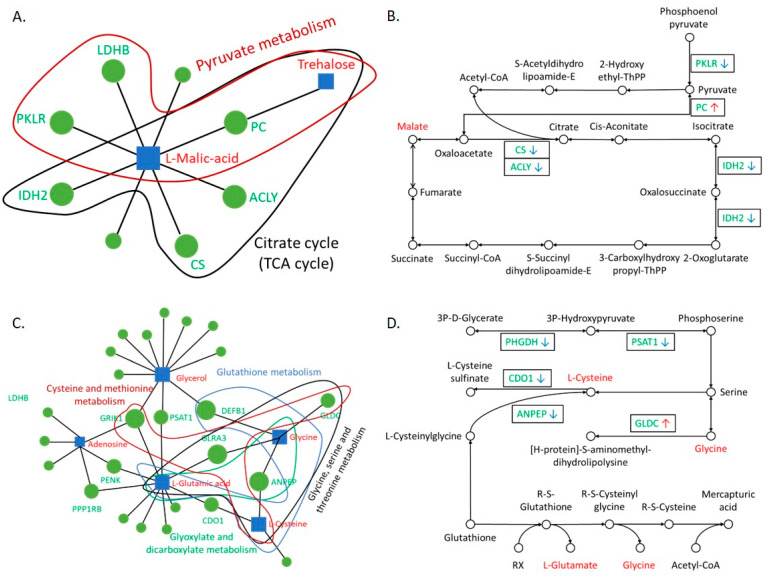
RNA–metabolite network analyses for liver (**A**) and heart (**C**) derived from heterologous compared to autologous mtDNA-supplemented pigs. Schematic diagram of pyruvate metabolism/citrate cycle (TCA cycle) in liver tissue (**B**) and glycine, serine and threonine metabolism/glutathione metabolism/cysteine and methionine metabolism in heart tissue (**D**). In A and C, the blue boxes indicate compounds and blue circles indicate mRNA. In C and D, circles and red font indicate compounds and blocks and green font indicate mRNA. Red and blue arrows indicate upregulated and downregulated gene expression in heterologous compared to autologous mtDNA-supplemented cohorts, respectively.

The mRNA–metabolite network analysis in the liver showed a connection between two metabolites, namely malic acid and trehalose, and eight genes. Some of those involved in pyruvate metabolism connected malic acid with pyruvate kinase L/R (*PKLR*), lactate dehydrogenase B (*LDHB*) and pyruvate carboxylase (PC), which overlapped with citrate metabolism connecting malic acid with PC, citrate synthase (CS), ATP citrate lyase (*ACLY*), and isocitrate dehydrogenase 2 (*IDH2*; Figure 4A). A schematic diagram mapping of the mRNA–metabolite interactions in the KEGG pathways is presented in Figure 4B.

In heart tissue, we found that differential mRNA–metabolite interactions affected several protein metabolism pathways, including glycine, serine and threonine metabolism, glutathione metabolism, cysteine and methionine metabolism, and glyoxylate and dicarboxylate metabolism (Table S12). Furthermore, we found a connection between five metabolites, namely glutamate, glycerol, adenosine, glycine and cysteine, and 25 genes. Some of those are involved in cysteine and methionine metabolism, including cysteine, glutamate ionotropic receptor AMPA type subunit 3 (*GLR3A*), glutamate ionotropic receptor kainate type subunit 1 (*GRIK1*) and glycine decarboxylase (*GLDC*); glutathione metabolism, including cysteine, glutamate and glycine with defensin beta 1 (*DEFB1*); glycine, serine and threonine metabolism, including cysteine, glutamate, and glycine with alanyl aminopeptidase (*ANPEP*) and *GLR3A*; and glyoxylate and dicarboxylate metabolism, including glutamate and glycine with *ANPEP* (Figure 4C). Schematic diagram mapping of the mRNA–metabolite interactions in the KEGG pathways is presented in Figure 4D. However, no gene–metabolite interaction networks were identified in brain tissue.

We did not find any indication that mtDNA supplementation (autologous and heterologous combined) affected OXPHOS metabolism. However, for the comparison between autologous and heterologous supplementation, we found that fumaric acid (fumarate) was the only metabolite that was differentially relatively abundant, and this was limited to the liver and heart. Fumarate is the residue of succinate oxidation, which takes place in subunit A of the succinate dehydrogenase complex (SDHA) and converts FAD into FADH2 during OXPHOS [34]. Regardless, the relative abundance of fumarate was inconsistent since it was relatively lower in the liver (Table 3) but higher in the heart (Table 4) for the heterologous-derived cohort.

## 4. Discussion

The metabolome describes small molecule metabolites (<1500 Da) found in a specific cell, tissue or body fluid [35]. As the downstream products of serial gene expression, translation and protein or enzyme modification, metabolites are the outputs of these biological processes [36]. In previous work, we found that autologous mtDNA supplementation of pig oocytes altered the DNA methylation and mRNA expression profiles of blastocyst-stage embryos [10] as well as the gene expression profiles of organs such as the brain, liver and heart in the cohort of pigs used here that had reached sexual maturity [22]. Here, we investigated the effect of mtDNA supplementation in these offspring and the effect of different sources of mtDNA for the supplementation process on the metabolite profiles of pig brain, liver and heart tissue. Furthermore, we observed the biological pathways affected by those metabolite changes and in combination with the transcriptome for each of the tissues. Whilst the pigs generated by mtDNA supplementation did not appear to have any gross physiological or biochemical/haematological differences once reaching adulthood, the metabolomic profile of individual tissues provides a much more detailed analysis of the overall metabolic health of the offspring.

We first compared the metabolite and the mRNA–metabolite network profiles of the brain, liver, and heart tissues derived from mtDNA supplementation per se against control offspring. We observed a limited number of relatively abundant metabolites as a result of mtDNA supplementation, especially in the brain (three metabolites) and liver (one metabolite) tissues (Table 1), and a low number of DEGs, especially in the brain (9 DEGs) and heart (15 DEGs) tissues (Table S1). These did not lead to any mRNA–metabolite networks in any of the tissues. In heart tissue derived from mtDNA-supplemented pigs, we found that relative abundances for stearic acid and elaidic acid were around two times higher compared to the control (*p* < 0.05), and both are involved in the biosynthesis of unsaturated fatty acids. Stearic acid is an 18-carbon chain-length saturated fatty acid that is commonly synthesised from animal fat or vegetable oil [37], whilst elaidic acid is an unsaturated trans-fatty acid that is strongly associated with cardiovascular disease [38].

Secondly, we compared metabolite profiles and mRNA–metabolite network profiles in brain, liver and heart tissues, which were supplemented with either autologous or heterologous mtDNA. We found that the relative abundance of mannose and its derivate, mannose-6-phosphate, as well as other monosaccharide derivates such as fructose-6-phosphate and glucose-6-phosphate in brains derived from heterologous mtDNA-supplemented pigs were around three times lower (*p* < 0.01) (Table 2). Those metabolites were involved in several carbohydrate metabolic pathways, including starch and sucrose metabolism, fructose and mannose metabolism, galactose metabolism, and amino sugar and nucleotide sugar metabolism (Table S2). Even though not statistically significant (*p* = 0.075), glucose relative abundance in brains derived from autologous mtDNA-supplemented pigs was around two times higher. As the highest energy-demanding organ in the body, the brain consumes ~20% of the body’s glucose and oxygen supply [26,39]. Since the brain’s ability to produce endogenous glucose falls behind its consumption, a constant glucose supply is critical to maintaining normal brain function as well as neuronal activity [26]. Low glucose levels in the brain, within the physiological range (3.9–6.1 mmol/L), can lead to decreased insulin secretion. If the glucose level falls just below the physiological range, for instance, 3.8 mmol/L, it will increase pancreatic β cell glucagon and adrenomedullary epinephrine secretion. When glucose levels decrease to 3.0 mmol/L, it triggers a more intense sympathoadrenal response that causes neurogenic (or autonomic) and neuroglycopenic symptoms. Further lower levels can cause aberrant behaviours, seizure, and coma [40]. Interestingly, our previous work indicated that blood glucose levels in heterologous mtDNA-supplemented pigs (4.2 mmol/L, range 3.7–4.5, n = 3) were relatively lower compared to autologous mtDNA-supplemented pigs (4.9 mmol/L, range 4.8–5.0, n = 2) [19]. Nonetheless, the difference was still within the normal blood glucose range for pigs (2.9–5.9 mmol/L) [41].

The liver is known for its function as a metabolic organ, which plays a critical role in receiving dietary nutrients and converting them into substrates that can be used, stored and re-converted when the cell or another organ requires them. This is related to another function of the liver, which maintains glucose homeostasis [42] by controlling the balance between glycogenesis and glycogenolysis [43]. We found that the carbohydrate compounds, including fumaric acid, malic acid, trehalose, maltose and cellobiose in liver derived from heterologous mtDNA-supplemented pigs were 1.41 to 2.70 times lower compared to those derived from autologous mtDNA-supplemented pigs (Table 3). These compounds were involved in starch and sucrose metabolism, the TCA cycle, and pyruvate metabolism (Table 3). Interestingly, despite lower carbohydrate metabolism, the relative abundance of glucose in the liver between heterologous and autologous mtDNA-supplemented tissues was similar. We argue that this is due to the liver’s ability to synthesise glucose through gluconeogenesis from other substrates, including, but not limited to, glutamine, fructose, lactate, alanine and glycerol [44]. Our findings support this notion. We found that glyceric acid, derived from glycerol, and tyrosine were relatively higher in liver derived from the heterologous cohort compared to the autologous group (Table 3).

Furthermore, the mRNA–metabolite network analysis for the liver showed the connection between malate and IDH2, CS, ACLY and PC genes in the TCA cycle. Additionally, we linked malate and PKLR, LDHB and PC genes in pyruvate metabolism (Figure 4A). The most recent review in liver energy supply suggested that during gluconeogenesis, oxaloacetate (OA) is transported from the mitochondria to the cytosol in the form of malate [45], likely through the malate–aspartate shuttle. ACLY is an enzyme that cleaves citrate into OA and acetyl-CoA, and its expression is activated by lipopolysaccharides [46]. Furthermore, a clinical study showed that *ACLY* gene expression was correlated with lipid accumulation, oxidative damage and the secretion of inflammatory cytokines in TNFα-triggered human hepatocytes [47]. Therefore, inhibition of *ACLY* can be used to improve non-alcoholic steatohepatitis (NASH), liver fibrosis and dyslipidaemia [48].

In addition, PC is the enzyme that converts pyruvate to OA [46]. The expression of PC in the liver plays a pivotal role in maintaining oxidation, biosynthesis, and pathways distal to the TCA cycle, whilst low expression of PC decreased aspartate and compromised urea cycle function, causing elevated urea cycle intermediates and hyperammonemia [49]. The schematic diagram in Figure 4B highlights how the downregulation of *CS*, *ACLY*, *IDH2* and *PKLR*, and the upregulation of PC lead to a lower abundance of malate in liver tissues derived from the heterologous mtDNA-supplemented cohort.

Due to its activity, the heart is a high-energy demanding organ that needs a constant supply of ATP to maintain cell activity, including normal cell function, growth and contraction [50]. Under normal physiological conditions, the heart produces its ATP from the oxidation of fatty acid (60% to 70% of total ATP) and oxidation of glucose (30% to 40%) and other substrates, including lactate, amino acids and ketone bodies [51]. We found that the different sources of mtDNA used for supplementation resulted in 14 differentially relatively abundant metabolites (Table 4), with seven metabolites found to be relatively higher in hearts derived from heterologous mtDNA-supplemented pigs. Pathway analysis showed mostly protein metabolism was significantly affected by those compounds, including glutathione metabolism; arginine biosynthesis; pentose and glucuronate interconversions; pantothenate and CoA biosynthesis; alanine, aspartate and glutamate metabolism; glyoxylate and dicarboxylate metabolism; porphyrin metabolism; and glycine, and serine and threonine metabolism (Table S4). Those pathways highlighted glutamate, cysteine, glycine, pantothenate and fumarate in the schematic figure of interconnected pathways (Figure 3). Furthermore, joint pathway analysis narrowed down the significantly different pathways (*p* < 0.05; minimum hit = 3) to glycine, serine and threonine metabolism; glutathione metabolism; cysteine and methionine metabolism; and glyoxylate and dicarboxylate metabolism (Table S12). It also highlighted the interconnections between glutamate, cysteine and glycine with the expression of *PHGDH*, *GLR3*, *DEFB1*, *ANPEP*, *GLDC* and *GRIK1* (Figure 4B). Physiologically, heart contraction is triggered by both spontaneous and periodical sinoatrial node pacemaker cells, which is referred to as spontaneous firing [52], and is generated by a unique cellular activity called local Ca^2+^ release. A recent study using a mouse model showed that glutamate in the heart plays a pivotal role in the initiation of spontaneous firing [53]. In addition, in a transverse aortic constriction (TAC)-induced cardiac hypertrophy rat model, glycine administration prevented pressure overload induced by cardiac hypertrophy [54], whilst in a clinical study, plasma glycine was negatively correlated with acute myocardial infarction [55]. Information regarding *PHGDH*, *CDO1* and *GLDC* gene expression in relation to heart function is limited. However, *PDGDH* [56] and GLDC [57] are overexpressed in many human cancer cells [56], whilst *CDO1* is a known tumour suppressor gene in many human cancer cells [58,59]. However, *PHGDH* knockout mice presented with decreased intracellular glutathione [60], which demonstrates its protective role as an antioxidant. Nonetheless, the schematic diagram (Figure 4D) highlighted the mechanisms of how the relatively less abundant glutamate, cysteine and glycine in the heart derived from heterologous mtDNA supplementation were modulated by downregulation of *PHGDH*, *CDO1*, *ANPEP* and *PSAT1*, and upregulation of *GLDC* gene expression. Taken together, mtDNA supplementation may compromise heart function.

Although we observed differences between the sources of mtDNA used in the supplementation process, only ~780 copies of mtDNA were introduced into oocytes [19] which would normally possess >200,000 copies of mtDNA [1]. Given the low amount of the supplement introduced, it is highly unlikely that the supplemented copies would produce extra units of energy, especially as OXPHOS pathway representation did not appear to be affected in our outcomes. It is more likely that the supplement induced a series of epigenomic and genomic events [61].

In previous work, we have shown that autologous mtDNA supplementation induces an mtDNA replication event that takes place at the two-cell stage of embryo development, which results in a large turnover of mtDNA [2] that alters the balance between the two genomes (nuclear and mitochondrial) [2,10]. This results in changes to DNA methylation and gene expression by the blastocyst stage of development [2,10] which, in turn, acts on tissue gene expression and metabolism. Consequently, the early effects are transmissible to the offspring. These effects are concerning if this form of technology is applied to patients with poor oocyte quality, where the effects could manifest to a greater extent. These oocytes likely have larger-scale molecular aberrations that could have a significant impact.

In terms of heterologous supplementation, other problems may manifest through, for example, innate immunity responses and the inflammasome [62]. Indeed, it has been argued that the mitochondrial genome is considered by the cell to be a ‘foreign’ genome and induces responses through, for example, the cGAS–STING and AIM2 pathways [62,63]. These pathways are likely active when mtDNA is released from the mitochondrion and/or the cell. It is also argued that mtDNA is expelled from the oocyte during in vitro culture [64]. Whether this primes the endometrium for receptivity remains to be clarified. Although autologous sources of mtDNA might be recognised as foreign, third-party (heterologous) mtDNA would receive greater recognition by the developing embryo and the mother as foreign. Coupled with this, we know that the metabolome is affected by immune responses [65], and, to this effect, we see a switch in the brain and liver to glycolysis-driven metabolic pathways. As a result, it is likely that sexually mature offspring are primed for potential autoimmune responses later in life, which might be more prevalent in heterologous-derived offspring. Interestingly, the heterologous offspring exhibited homoplasmy at a 2% threshold [19], which suggests that any effects are responses immediate to the supplementation process and are programmed throughout life. Furthermore, whilst it has been suggested that 1% heteroplasmy can lead to metabolic disruption [18], our work suggests that as little as 0.39% mtDNA introduced by supplementation is sufficient to lead to this effect. This might be reliant on the single mtDNA replication event that takes in the embryo prior to gastrulation at the two-cell stage [2] to mediate a response and would be dependent on the efficiency of processes to activate mitophagy pathways which remove and recycle damaged mitochondria and promote the biogenesis of new, fully functional mitochondria in the oocyte and embryo [66], as is the case for degradation of paternal mtDNA [67].

These changes in metabolism may also result from compensatory or trade-off mechanisms that have been observed in other species [68,69]. In previous work, using a mouse model, we observed a trade-off between increased fertility and a cardiac disorder that was present transgenerationally [70]. The female offspring exhibited differences in gene expression in primordial follicles and possessed higher ovarian reserves and larger litter sizes. Indeed, in a number of species, fertility has been linked to trade-offs with metabolism [71]. While we did not observe similar trade-offs in our pig model, it is feasible that others could arise through effects on innate immunity and the inflammasome.

Whilst much interest has been centred on mitochondrial function and mtDNA copy number in the oocyte in terms of fertilisation outcome, perhaps it is appropriate to suggest an association between mtDNA copy number in the oocyte and adult metabolic health. To this extent, it is feasible that offspring derived from the lower end of the mtDNA spectrum associated with fertilisation success might, indeed, exhibit symptoms similar to the disorders associated with mtDNA depletion [72] whilst those with higher levels may be healthier. It is interesting to note that in previous studies, we did not record increased blastocyst development rates for oocytes with the requisite numbers of mtDNA copies when undergoing supplementation [2]. Increased blastocyst rates were only recorded for those oocytes with depleted levels. Consequently, the mature fertilisable oocyte has its population of mtDNA set for subsequent developmental events and would withstand the reduction in mtDNA copy that takes place prior to gastrulation [2,7,8,64] and have sufficient present post-gastrulation to populate cells with mtDNA in a cell-specific manner [73]. However, simply adding extra copies of mtDNA into a mature fertilisable oocyte perturbs the genomic balance established during oogenesis and alters gene expression and cellular metabolism. Added to this, metabolites associated with the TCA cycle are modulated, which, in turn, could affect the epigenetic status of the respective tissue through DNA methylation [74] and histone [75] modifications.

## 5. Conclusions

From our findings, we conclude that mtDNA supplementation in a pig model at sexual maturity, regardless of the source of supplemented mtDNA, altered the metabolic profiles and mRNA expression in the brain, liver and heart. The number of differentially abundant metabolites was greater in the comparison between heterologous and autologous mtDNA supplementation compared to overall mtDNA supplementation against the control. In the overall analysis of mtDNA supplementation against the control, we found that stearic acid and elaidic acid, which are involved in the biosynthesis of unsaturated fatty acids, were less abundant in the mtDNA-supplemented cohort. In addition, heterologous mtDNA supplementation altered numerous forms of carbohydrate metabolism in the brain and liver, including fructose and mannose metabolism and starch and sucrose metabolism, respectively. In the heart, protein metabolism, especially glutathione metabolism, is the most affected pathway. mRNA–metabolite analysis showed how low abundance of malate in the liver and glutamate, cysteine and glycine in the heart derived from the heterologous mtDNA-supplemented cohort was controlled by downregulation of their associated gene expression, including *CS*, *ACLY* and *IDH2* in the liver and *PHGDH*, *PSAT1*, *ANPEP* and *CDO1* in the heart. Taken together, our findings suggest that mtDNA supplementation modulates the metabolome and transcriptome of the tissues of sexually mature offspring. However, it appears that the use of autologous mtDNA is preferable as a source for mtDNA supplementation than heterologous mtDNA, given that the changes observed were less remarkable.

## Figures and Tables

**Table 1:** Differentially relatively abundant compounds in brain, liver and heart tissues derived from all mtDNA-supplemented pigs compared to control pigs.

Tissue	Compound	Macromolecule Group	t.Stat	*p*.Value	FC
Brain	2-Hydroxyisobutyric acid	Carbohydrate	−6.7006	0.000277	1.4629
	5-Oxoproline	Protein	2.5323	0.039099	0.8593
	Stearic acid	Lipid	−2.4387	0.044847	1.2098
Liver	Inositol	Carbohydrate	−3.0481	0.018633	1.2447
Heart	Ribose 5-phosphate	Carbohydrate	6.0894	0.000496	0.7299
	N-Acetylglucosamine	Carbohydrate	−3.3097	0.012947	1.6453
	Stearic acid	Lipid	−3.1869	0.015343	1.8134
	Threonine	Protein	−2.9181	0.022401	1.4837
	2-Hydroxyisobutyric acid	Carbohydrate	−2.6224	0.034288	1.6318
	Margaric acid	Lipid	−2.5935	0.035762	1.6861
	Hypotaurine	Carbohydrate	2.5212	0.039741	0.6628
	Creatinine	Protein	2.4938	0.041368	0.7299
	Elaidic acid	Lipid	−2.4266	0.045646	2.3195
	Oleic acid	Lipid	−2.4228	0.045904	2.2863
	Monostearin	Lipid	−2.4144	0.046469	1.6117

FC: Fold change.

**Table 2:** Differentially relatively abundant compounds in brain tissue derived from heterologous compared to autologous mtDNA-supplemented pigs.

Compound	Macromolecule Group	t.Stat	*p*.Value	FC
Mannose 6-phosphate (2)	Carbohydrate	9.2363	0.000764	0.3088
Fructose 6-phosphate	Carbohydrate	8.3014	0.00115	0.3253
2-Aminoethanol *	Carbohydrate	−7.352	0.001823	1.5917
Mannose	Carbohydrate	6.1057	0.003642	0.4307
Glucose 6-phosphate	Carbohydrate	5.9169	0.004086	0.3095
Mannose 6-phosphate (1)	Carbohydrate	5.698	0.004688	0.3094
Ornithine	Protein	−4.0231	0.015824	1.0997
3-Phosphoglyceric acid	Carbohydrate	−3.4666	0.025663	3.9154
Galactose	Carbohydrate	3.4269	0.026612	0.2214
Uracil	Nucleic acid	−3.1232	0.035417	1.2482
Thymine	Nucleic acid	−2.9307	0.042783	1.5650

FC: Fold change. Mannose 6-phosphate (1) and (2) are stereoisomers. * exact match with glutamate by MetaboAnalyst.

**Table 3:** Differentially relatively abundant compounds in liver tissue derived from heterologous compared to autologous mtDNA-supplemented pigs.

Compound	Macromolecule Group	t.Stat	*p*.Value	FC
Fumaric acid	Carbohydrate	9.1781	0.000783	0.6055
Glyceric acid	Carbohydrate	−8.2775	0.001163	1.4720
Malic acid	Carbohydrate	6.8237	0.002412	0.7109
Arabitol	Carbohydrate	3.7916	0.019243	0.4753
1-Hexadecanol	Lipid	−3.6086	0.022585	1.2286
Valine	Protein	−3.4966	0.024973	1.3982
Inositol	Carbohydrate	3.1921	0.033151	0.9034
Lactitol	Carbohydrate	3.123	0.035422	0.2996
Trehalose	Carbohydrate	3.1118	0.03581	0.3675
Cellobiose	Carbohydrate	3.038	0.038477	0.3695
Maltose	Carbohydrate	3.0334	0.038653	0.3759
Tyrosine	Protein	−3.0138	0.039404	1.4154

FC: Fold change.

**Table 4:** Differentially relatively abundant compounds in heart tissue derived from heterologous compared to autologous mtDNA-supplemented pigs.

Compound	Macromolecule Group	t.Stat	*p*.Value	FC
Creatinine	Protein	−5.1338	0.00682	1.2854
Palmitic acid	Lipid	4.5255	0.010614	0.6294
Glycerol	Carbohydrate	−3.9877	0.016297	1.6960
Glycine	Protein	3.6678	0.021432	0.8244
Margaric acid	Lipid	3.575	0.023272	0.6932
Fumaric acid	Carbohydrate	−3.4668	0.025659	1.7649
Ribulose	Carbohydrate	3.2162	0.032396	0.4800
Glutamate	Protein	3.182	0.03347	0.5841
Cysteine	Protein	2.9895	0.040358	0.4758
3-Hydroxyisovaleric acid	Carbohydrate	−2.8749	0.045245	2.0432
Pantothenic acid	Protein	−2.851	0.046349	1,5528
Adenosine	Nucleic acid	−2.8138	0.04813	2.1796
Lactic acid	Carbohydrate	−2.7991	0.048857	1.1722
Xylulose	Carbohydrate	2.7815	0.049741	0.5058

FC: Fold change.

## Data Availability

Data for the DEG analysis are publicly available under the BioProject ID PRJNA823749. The metabolite data that support the findings of this study are available in this manuscript. However, should raw data be needed, they are available from the corresponding author upon reasonable request. Data are located in secured access data storage at the University of Adelaide.

## Supplementary Materials

The following supporting information can be downloaded at: https://www.mdpi.com/article/10.3390/biom14111477/s1, Figure S1. Data distribution after normalisation and scaling. Data normalisation was performed using probabilistic quotient normalisation (PQN), and auto-scaling was performed by dividing each variable with the standard deviation of each variable in the control cohort; Figure S2. Heatmaps illustrating fold change of the top 25 metabolites in brain (A), liver (B) and heart (C) derived from all supplemented pigs compared to control; Figure S3. Heatmaps illustrating fold change of the top 25 metabolites in brain (A), liver (B) and heart (C) derived from heterologous mtDNA-supplemented pigs compared to autologous mtDNA-supplemented pigs; Table S1: Enriched KEGG metabolic pathways using differentially relative abundance of metabolites in brain, liver and heart tissues derived from all mtDNA-supplemented compared to control pigs; Table S2. Enriched KEGG metabolic pathways using differentially relative abundance of metabolites in brain tissue derived from heterologous compared to autologous mtDNA-supplemented pigs; Table S3. Enriched KEGG metabolic pathways using differentially relative abundance of metabolites in liver tissue derived from heterologous compared to autologous mtDNA-supplemented pigs; Table S4. Enriched metabolism KEGG pathways using differentially relative abundance of metabolites in heart tissue derived from heterologous compared to autologous mtDNA-supplemented pigs; Table S5. The list of differentially expressed genes in the brain derived from mtDNA-supplemented compared to control pigs with *p*-value < 0.05 and false discovery rate (FDR) < 0.05; Table S6. The list of differentially expressed genes in the liver derived from mtDNA-supplemented compared to control pigs with *p*-value < 0.05 and false discovery rate (FDR) < 0.05; Table S7. The list of differentially expressed genes in the heart derived from mtDNA-supplemented compared to control pigs with *p*-value < 0.05 and false discovery rate (FDR) < 0.05; Table S8. The list of differentially expressed genes in the brain derived from heterologous mtDNA-supplemented compared to autologous cohort pigs with *p*-value < 0.05 and false discovery rate (FDR) < 0.05; Table S9. The list of differentially expressed genes in the liver derived from heterologous mtDNA-supplemented compared to autologous cohort pigs with *p*-value < 0.05 and false discovery rate (FDR) < 0.05; Table S10. The list of differentially expressed genes in the heart derived from heterologous mtDNA-supplemented compared to autologous cohort pigs with *p*-value < 0.05 and false discovery rate (FDR) < 0.05; Table S11. Enriched metabolism KEGG pathways using RNA–metabolite networks in offspring liver tissues derived from heterologous compared to autologous mtDNA-supplemented pigs; Table S12. Enriched KEGG metabolic pathways using RNA–metabolite networks in offspring heart tissues derived from heterologous compared to autologous mtDNA-supplemented pigs.
