# Supplementary material for: Supplementation of Oocytes by Microinjection with Extra Copies of mtDNA Alters Metabolite Profiles and Interactions with Expressed Genes in a Tissue-Specific Manner
Source: Biomolecules. 2024 Nov 20;14(11):1477. doi: 10.3390/biom14111477 (PMC11591607; doi:10.3390/biom14111477)
Supplement: Supplementary file 1 [file biomolecules-14-01477-s001.zip › Supplementary Information_Andreas et al.pdf]

SUPPLEMENTARY INFORMATION

Supplementation of oocytes by microinjection with extra copies of mtDNA alters metabolite profiles and interactions with expressed genes in a tissue specific manner.

Eryk Andreas [1], Alexander Penn [1], Takashi Okada [1] and Justin C. St. John [1,*]

[1] Experimental Mitochondrial Genetics Group, School of Biomedicine, Faculty of Health and Medical Sciences, The University of Adelaide, Adelaide Health and Medical Sciences Building, Adelaide, SA 5000, Australia; eryk.andreas@adelaide.edu.au; alexander.penn@adelaide.edu.au; takashi.okada@adelaide.edu.au

* Correspondence: jus.stjohn@adelaide.edu.au

Table S1. Enriched KEGG metabolic pathways using differentially relative abundance of metabolites in brain, liver and heart tissues derived from all mtDNA supplemented compared to control pigs.

Tissue	KEGG Pathways	total	expected	hits	Raw p
Brain	Glutathione metabolism	28	0.0364	1	0.0361
	Biosynthesis of unsaturated fatty acids	36	0.0468	1	0.0463
Liver	Ascorbate and aldarate metabolism	9	0.00585	1	0.00585
	Galactose metabolism	27	0.0175	1	0.0175
	Inositol phosphate metabolism	30	0.0195	1	0.0195
Heart	Biosynthesis of unsaturated fatty acids	36	0.14	2	0.00753
	Valine, leucine and isoleucine biosynthesis	8	0.0312	1	0.0308
	Taurine and hypotaurine metabolism	8	0.0312	1	0.0308
	Pentose phosphate pathway	23	0.0897	1	0.0865
	Glycine, serine and threonine metabolism	33	0.129	1	0.122
	Amino sugar and nucleotide sugar metabolism	42	0.164	1	0.153
	Purine metabolism	70	0.273	1	0.244

Table S2. Enriched KEGG metabolic pathways using differentially relative abundance of metabolites in brain tissue derived from heterologous compared to autologous mtDNA supplemented pigs.

KEGG Pathway	total	expected	hits	Raw p
Fructose and mannose metabolism	20	0.117	3	0.00015
Amino sugar and nucleotide sugar metabolism	42	0.246	3	0.00142
Arginine biosynthesis	14	0.0819	2	0.00267
Starch and sucrose metabolism	18	0.105	2	0.00443
Galactose metabolism	27	0.158	2	0.00989
Glutathione metabolism	28	0.164	2	0.0106
Neomycin, kanamycin and gentamicin biosynthesis	2	0.0117	1	0.0117
Arginine and proline metabolism	36	0.211	2	0.0173
Pyrimidine metabolism	39	0.228	2	0.0201
Nitrogen metabolism	6	0.0351	1	0.0346
Butanoate metabolism	15	0.0877	1	0.0846
Histidine metabolism	16	0.0936	1	0.09
Pantothenate and CoA biosynthesis	20	0.117	1	0.111
β-Alanine metabolism	21	0.123	1	0.117
Pentose phosphate pathway	23	0.135	1	0.127
Glycolysis / Gluconeogenesis	26	0.152	1	0.143
Alanine, aspartate and glutamate metabolism	28	0.164	1	0.153
Inositol phosphate metabolism	30	0.175	1	0.163
Glyoxylate and dicarboxylate metabolism	31	0.181	1	0.168
Porphyrin metabolism	31	0.181	1	0.168

Table S3. Enriched KEGG metabolic pathways using differentially relative abundance of metabolites in liver tissue derived from heterologous compared to autologous mtDNA supplemented pigs.

KEGG Pathway	total	expected	hits	Raw p
Starch and sucrose metabolism	18	0.105	3	0.000108
Citrate cycle (TCA cycle)	20	0.117	2	0.00547
Pyruvate metabolism	23	0.135	2	0.00722
Glyoxylate and dicarboxylate metabolism	31	0.181	2	0.0129
Tyrosine metabolism	42	0.246	2	0.0232
Phenylalanine, tyrosine and tryptophan biosynthesis	4	0.0234	1	0.0232
Valine, leucine and isoleucine biosynthesis	8	0.0468	1	0.0459
Phenylalanine metabolism	8	0.0468	1	0.0459
Ascorbate and aldarate metabolism	9	0.0526	1	0.0515
Arginine biosynthesis	14	0.0819	1	0.0792
Glycerolipid metabolism	16	0.0936	1	0.09
Ubiquinone and other terpenoid-quinone biosynthesis	18	0.105	1	0.101
Pantothenate and CoA biosynthesis	20	0.117	1	0.111
Pentose phosphate pathway	23	0.135	1	0.127
Galactose metabolism	27	0.158	1	0.148
Alanine, aspartate and glutamate metabolism	28	0.164	1	0.153
Inositol phosphate metabolism	30	0.175	1	0.163
Glycine, serine and threonine metabolism	33	0.193	1	0.178
Valine, leucine and isoleucine degradation	39	0.228	1	0.207

Table S4. Enriched metabolism KEGG pathways using differentially relative abundance of metabolites in heart tissue derived from heterologous compared to autologous mtDNA supplemented pigs.

KEGG Pathway	total	expected	hits	Raw p
Glutathione metabolism	28	0.182	3	0.000595
Arginine biosynthesis	14	0.091	2	0.00332
Pentose and glucuronate interconversions	19	0.123	2	0.00613
Pantothenate and CoA biosynthesis	20	0.13	2	0.00679
Alanine, aspartate and glutamate metabolism	28	0.182	2	0.0131
Glyoxylate and dicarboxylate metabolism	31	0.201	2	0.016
Porphyrin metabolism	31	0.201	2	0.016
Glycine, serine and threonine metabolism	33	0.214	2	0.018
Nitrogen metabolism	6	0.039	1	0.0384
Thiamine metabolism	7	0.0455	1	0.0447
Taurine and hypotaurine metabolism	8	0.052	1	0.0509
Butanoate metabolism	15	0.0975	1	0.0936
Histidine metabolism	16	0.104	1	0.0995
Glycerolipid metabolism	16	0.104	1	0.0995
Citrate cycle (TCA cycle)	20	0.13	1	0.123
Pyruvate metabolism	23	0.149	1	0.14
Galactose metabolism	27	0.175	1	0.163
Lipoic acid metabolism	28	0.182	1	0.168
Cysteine and methionine metabolism	33	0.214	1	0.195
Arginine and proline metabolism	36	0.234	1	0.211
Biosynthesis of unsaturated fatty acids	36	0.234	1	0.211
Fatty acid elongation	38	0.247	1	0.222
Fatty acid degradation	39	0.253	1	0.227
Tyrosine metabolism	42	0.273	1	0.242
Primary bile acid biosynthesis	46	0.299	1	0.262

Fatty acid biosynthesis	47	0.305	1	0.267
Purine metabolism	70	0.455	1	0.373

Table S5. The list of differentially expressed genes in brain derived from mtDNA supplemented compared to control pigs with p-value < 0.05 and false discovery rate (FDR) < 0.05.

Gene	logFC	logCPM	LR	PValue	FDR
LOC110260659	-3.43145	4.31263	241.437	1.91E-54	3.57E-50
LOC100518848	-2.74717	0.90337	79.8133	4.12E-19	3.84E-15
LRAT	-2.82886	3.14343	29.6015	5.31E-08	0.00033
LOC110257650	-1.39084	1.73976	25.0345	5.63E-07	0.00263
LOC110258138	-2.03088	-0.68274	22.5015	2.10E-06	0.00784
LOC102163816	-1.69807	4.31494	19.4772	1.02E-05	0.02752
LOC102159834	-3.64499	1.39951	19.3762	1.07E-05	0.02752
LOC102157770	-3.17793	-0.37739	19.0703	1.26E-05	0.02752
SGCA	1.26476	4.64834	18.9722	1.33E-05	0.02752

Table S6. The list of differentially expressed genes in liver derived from mtDNA supplemented compared to control pigs with p-value < 0.05 and false discovery rate (FDR) < 0.05.

See Supplementary File

Table S7. The list of differentially expressed genes in heart derived from mtDNA supplemented compared to control pigs with p-value < 0.05 and false discovery rate (FDR) < 0.05.

Gene	logFC	logCPM	LR	PValue	FDR
LOC110260659	-2.93888	4.31263	202.548	5.81E-46	1.08E-41
LOC100518848	-2.36839	0.90337	61.606	4.20E-15	3.92E-11
LOC110260329	4.89304	-1.60893	57.7724	2.94E-14	1.83E-10
CHRNA4	-4.03243	1.29467	45.3279	1.67E-11	7.78E-08
LOC110261992	4.87873	1.24442	29.1761	6.61E-08	0.00021
PLIN5	-1.8192	3.7842	29.1249	6.79E-08	0.00021
MYT1	-2.84299	2.13888	25.0458	5.60E-07	0.00149
TMEM159	-1.4706	2.9625	24.1262	9.02E-07	0.00211
ABCC6	-2.4647	7.59454	19.9132	8.10E-06	0.01681
PDZD9	0.99394	2.5976	19.4443	1.04E-05	0.01934
SLC22A1	-4.10162	7.15982	19.2011	1.18E-05	0.01997
VARS2	-0.40867	5.05644	18.1241	2.07E-05	0.03221
LOC106505031	1.54572	-0.4945	17.4209	3.00E-05	0.04171
TTC39A	-1.29617	1.52662	17.2234	3.32E-05	0.04171
DAO	-3.15568	6.50528	17.2077	3.35E-05	0.04171

Table S8. The list of differentially expressed genes in brain derived from heterologous mtDNA supplemented compared to autologous cohort pigs with p-value < 0.05 and false discovery rate (FDR) < 0.05.

See Supplementary File

Table S9. The list of differentially expressed genes in liver derived from heterologous mtDNA supplemented compared to autologous cohort pigs with p-value < 0.05 and false discovery rate (FDR) < 0.05.

See Supplementary File

Table S10. The list of differentially expressed genes in heart derived from heterologous mtDNA supplemented compared to autologous cohort pigs with p-value < 0.05 and false discovery rate (FDR) < 0.05.

See Supplementary File

Table S11. Enriched metabolism KEGG pathways using RNA-metabolite networks in offspring liver tissues derived from heterologous compared to autologous mtDNA supplemented pigs.

KEGG Pathway	Total	Expected	Hits	p.value
Citrate cycle (TCA cycle)	42	0.172	5	2.80E-07
Pyruvate metabolism	45	0.184	4	1.96E-05
Glutathione metabolism	56	0.23	2	0.0207
Glyoxylate and dicarboxylate metabolism	56	0.23	2	0.0207
Glycolysis or Gluconeogenesis	61	0.25	2	0.0243
Starch and sucrose metabolism	43	0.176	1	0.163
Pentose phosphate pathway	47	0.193	1	0.177
Propanoate metabolism	48	0.197	1	0.18
Cysteine and methionine metabolism	71	0.291	1	0.256
Amino sugar and nucleotide sugar metabolism	79	0.324	1	0.281

Table S12. Enriched KEGG metabolic pathways using RNA-metabolite networks in offspring heart tissues derived from heterologous compared to autologous mtDNA supplemented pigs.

KEGG Pathway	Total	Expected	Hits	p.value
Glycine, serine and threonine metabolism	68	0.334	5	9.88E-06
Glutathione metabolism	56	0.275	4	0.000108
Cysteine and methionine metabolism	71	0.349	4	0.000273
Glyoxylate and dicarboxylate metabolism	56	0.275	3	0.00218
Taurine and hypotaurine metabolism	16	0.0787	2	0.00256
Aminoacyl-tRNA biosynthesis	74	0.364	3	0.00484
Porphyrin and chlorophyll metabolism	53	0.261	2	0.0266
D-Glutamine and D-glutamate metabolism	10	0.0492	1	0.0482
Nitrogen metabolism	10	0.0492	1	0.0482
Thiamine metabolism	14	0.0689	1	0.0669

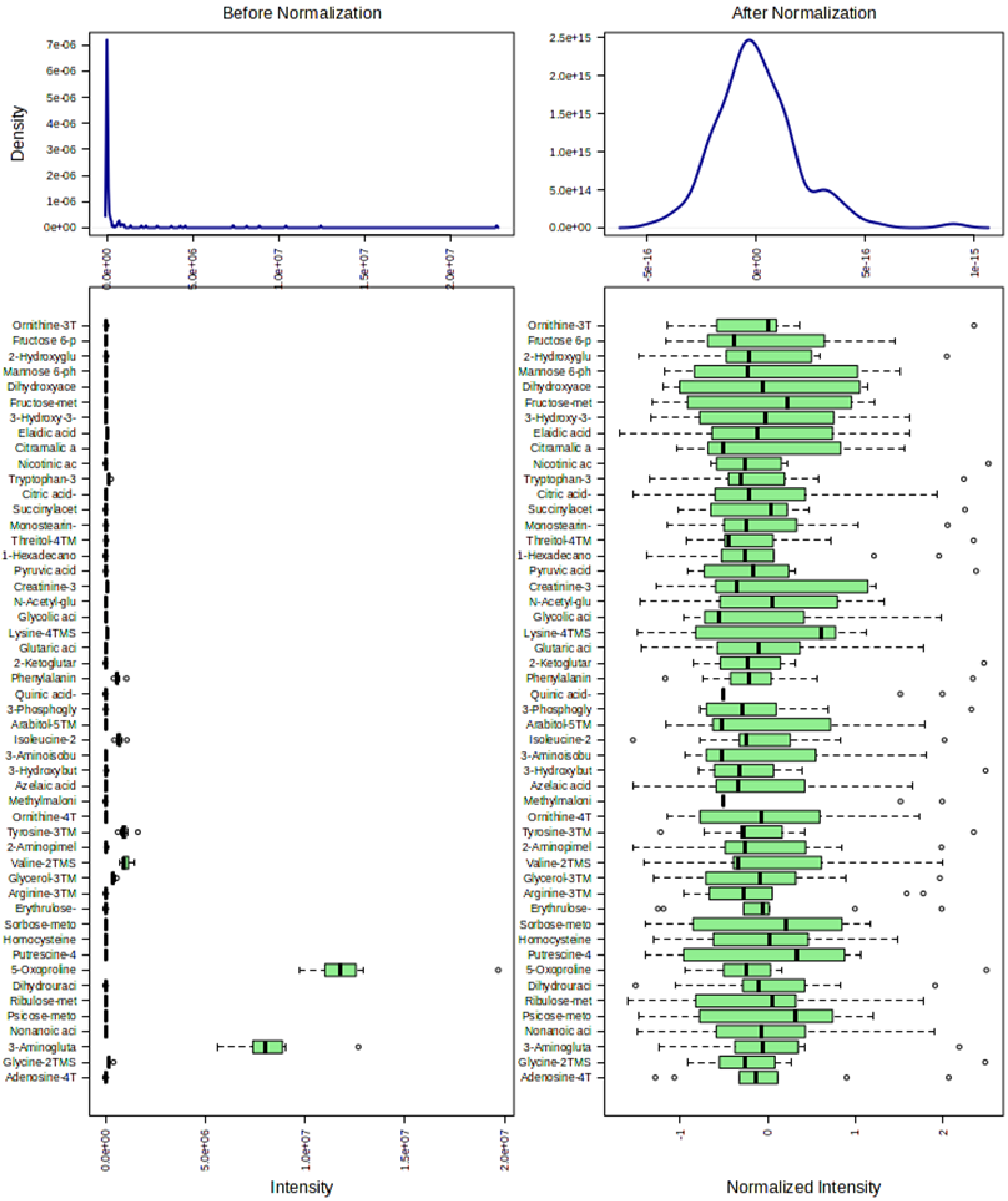

Figure S1. Data distribution after normalisation and scaling. Data normalisation was performed using probabilistic quotient normalization (PQN) and auto scaling was performed by dividing each variable with the standard deviation of each variable in the control cohort.

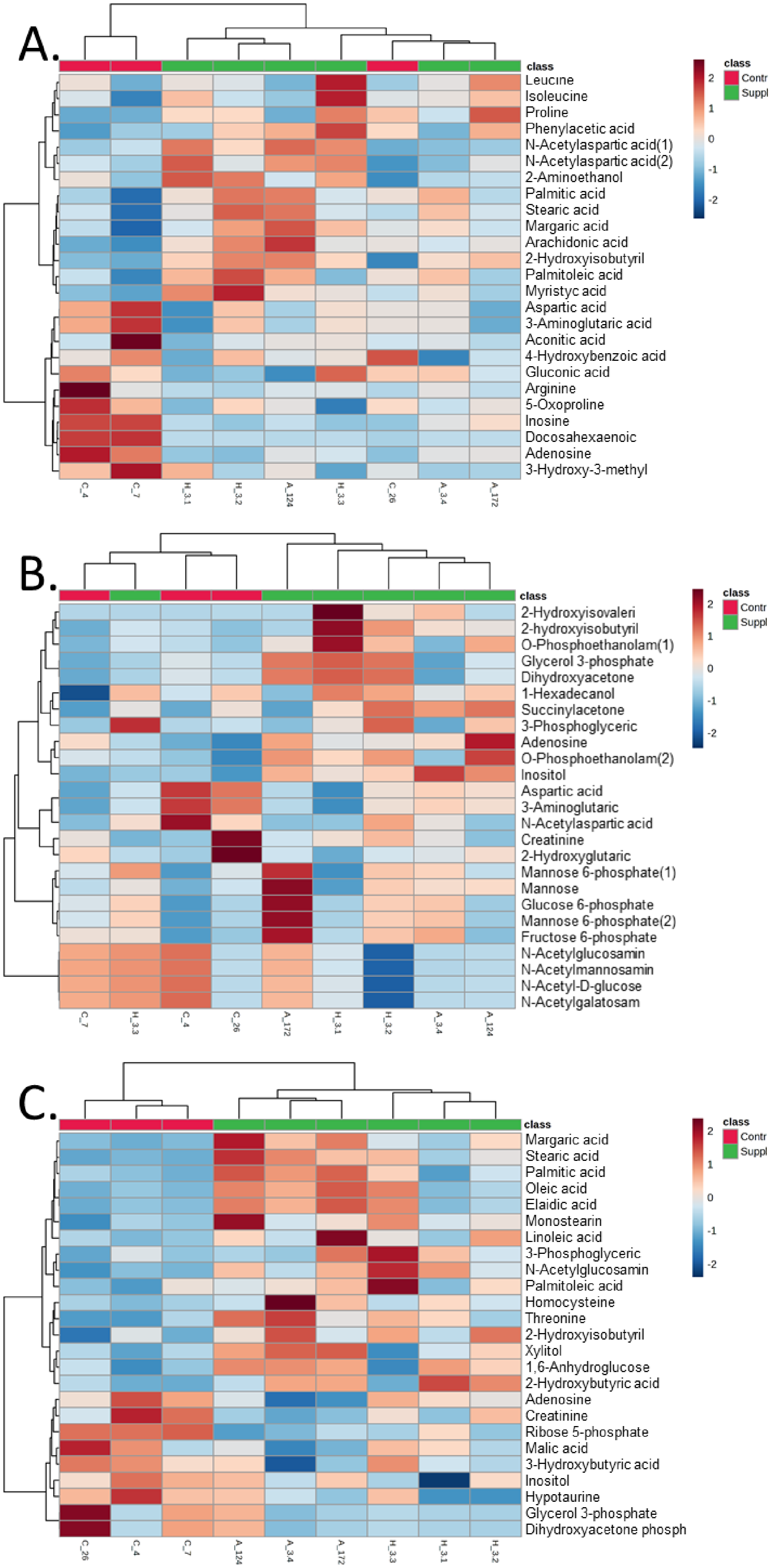
A.
class
Contr
Suppl
2
1
0
-1
-2
Leucine
Isoleucine
Proline
Phenylacetic acid
N-Acetylaspartic acid(1)
N-Acetylaspartic acid(2)
2-Aminoethanol
Palmitic acid
Stearic acid
Margaric acid
Arachidonic acid
2-Hydroxyisobutyril
Palmitoleic acid
Myristyc acid
Aspartic acid
3-Aminoglutaric acid
Aconitic acid
4-Hydroxybenzoic acid
Gluconic acid
Arginine
5-Oxoproline
Inosine
Docosahexaenoic
Adenosine
3-Hydroxy-3-methyl
C_4
C_7
H_3.1
H_3.2
A_124
H_3.3
C_26
A_3.4
A_172
B.
class
Contr
Suppl
2
1
0
-1
-2
2-Hydroxyisovaleri
2-hydroxyisobutyril
O-Phosphoethanolam(1)
Glycerol 3-phosphate
Dihydroxyacetone
1-Hexadecanol
Succinylacetone
3-Phosphoglyceric
Adenosine
O-Phosphoethanolam(2)
Inositol
Aspartic acid
3-Aminoglutaric
N-Acetylaspartic acid
Creatinine
2-Hydroxyglutaric
Mannose 6-phosphate(1)
Mannose
Glucose 6-phosphate
Mannose 6-phosphate(2)
Fructose 6-phosphate
N-Acetylglucosamin
N-Acetylmannosamin
N-Acetyl-D-glucose
N-Acetylgalatosam
C_7
H_3.3
C_4
C_26
A_172
H_3.1
H_3.2
A_3.4
A_124
C.
class
Contr
Suppl
2
1
0
-1
-2
Margaric acid
Stearic acid
Palmitic acid
Oleic acid
Elaidic acid
Monostearin
Linoleic acid
3-Phosphoglyceric
N-Acetylglucosamin
Palmitoleic acid
Homocysteine
Threonine
2-Hydroxyisobutyril
Xylitol
1,6-Anhydroglucose
2-Hydroxybutyric acid
Adenosine
Creatinine
Ribose 5-phosphate
Malic acid
3-Hydroxybutyric acid
Inositol
Hypotaurine
Glycerol 3-phosphate
Dihydroxyacetone phosph
C_26
C_4
C_7
A_124
A_3.4
A_172
H_3.3
H_3.1
H_3.2

Figure S2. Heatmaps illustrating fold change of the top 25 metabolites in brain (A), liver (B) and heart (C) derived from all supplemented pigs compared to control.

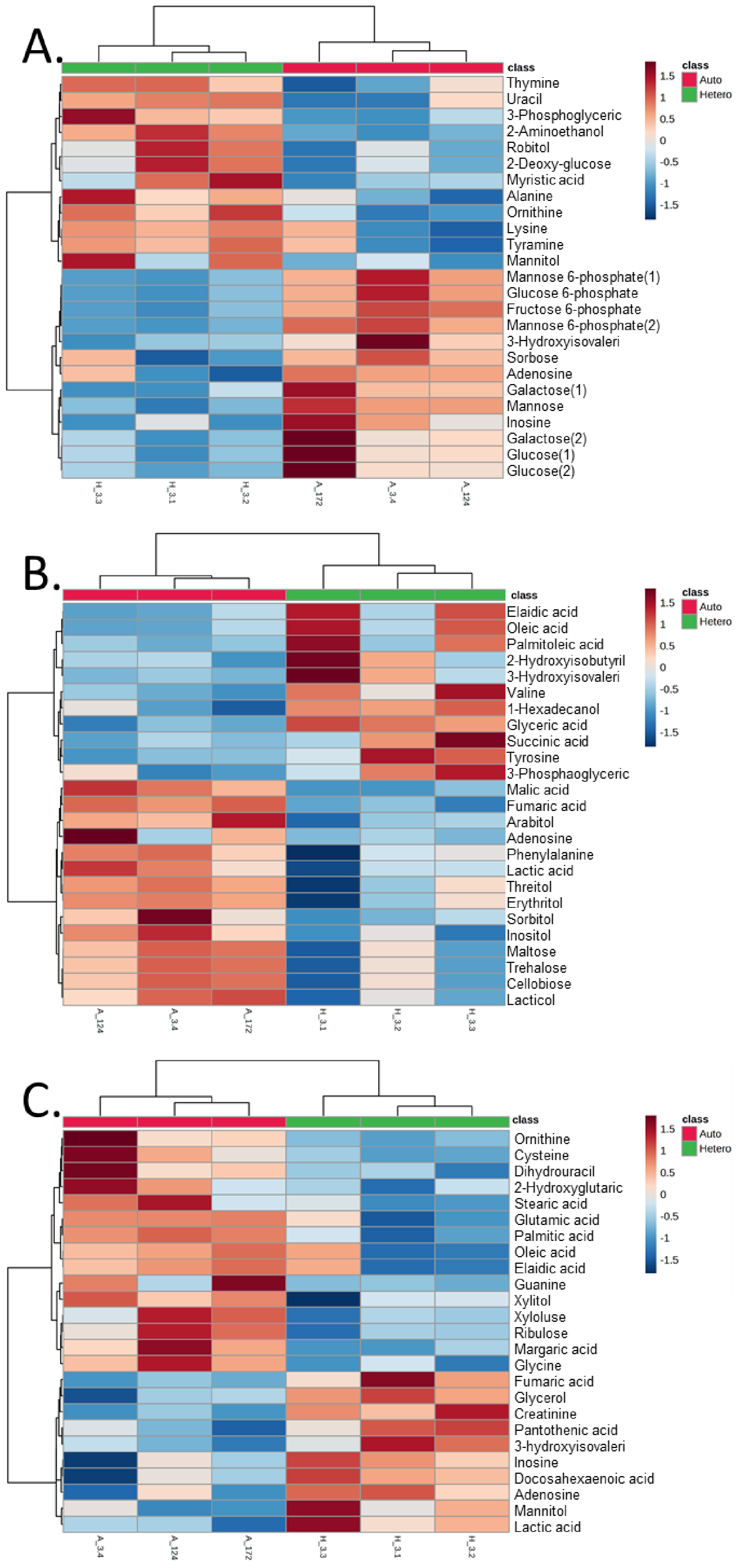
A.
class
Auto
Hetero
1.5
1
0.5
0
-0.5
-1
-1.5
Thymine
Uracil
3-Phosphoglyceric
2-Aminoethanol
Robitol
2-Deoxy-glucose
Myristic acid
Alanine
Ornithine
Lysine
Tyramine
Mannitol
Mannose 6-phosphate(1)
Glucose 6-phosphate
Fructose 6-phosphate
Mannose 6-phosphate(2)
3-Hydroxyisovaleri
Sorbose
Adenosine
Galactose(1)
Mannose
Inosine
Galactose(2)
Glucose(1)
Glucose(2)
H_3.3
H_3.1
H_3.2
A_172
A_3.4
A_124
B.
class
Auto
Hetero
1.5
1
0.5
0
-0.5
-1
-1.5
Elaidic acid
Oleic acid
Palmitoleic acid
2-Hydroxyisobutyril
3-Hydroxyisovaleri
Valine
1-Hexadecanol
Glyceric acid
Succinic acid
Tyrosine
3-Phosphaoglyceric
Malic acid
Fumaric acid
Arabitol
Adenosine
Phenylalanine
Lactic acid
Threitol
Erythritol
Sorbitol
Inositol
Maltose
Trehalose
Cellobiose
Lacticol
A_124
A_3.4
A_172
H_3.1
H_3.2
H_3.3
C.
class
Auto
Hetero
1.5
1
0.5
0
-0.5
-1
-1.5
Ornithine
Cysteine
Dihydrouracil
2-Hydroxyglutaric
Stearic acid
Glutamic acid
Palmitic acid
Oleic acid
Elaidic acid
Guanine
Xylitol
Xyloluse
Ribulose
Margaric acid
Glycine
Fumaric acid
Glycerol
Creatinine
Pantothenic acid
3-hydroxyisovaleri
Inosine
Docosahexaenoic acid
Adenosine
Mannitol
Lactic acid
A_3.4
A_124
A_172
H_3.3
H_3.1
H_3.2

Figure S3. Heatmaps illustrating fold change of the top 25 metabolites in brain (A), liver (B) and heart (C) derived from heterologous mtDNA supplemented pigs compared to autologous mtDNA supplemented pigs.
